# “A More Equitable Society”: The Politics of Global Fairness in Paralympic Sport

**DOI:** 10.1371/journal.pone.0167481

**Published:** 2016-12-12

**Authors:** Leslie Swartz, Jason Bantjes, Divan Rall, Suzanne Ferreira, Cheri Blauwet, Wayne Derman

**Affiliations:** 1 Alan J. Flisher Centre for Public Mental Health, Department of Psychology, Stellenbosch University, Stellenbosch, South Africa; 2 Department of Psychology, Stellenbosch University, Stellenbosch, South Africa; 3 Department of Sport Science, Stellenbosch University, Stellenbosch, South Africa; 4 Department of Physical Medicine and Rehabilitation, Spaulding Rehabilitation Hospital and Brigham and Women’s Hospital, Harvard Medical School, Boston, Massachusetts, United States of America; 5 Institute for Sport and Exercise Medicine, Stellenbosch University, Cape Town, South Africa and International Olympic Committee (IOC) Research Centre, Cape Town, South Africa; Universita degli Studi di Perugia, ITALY

## Abstract

The Paralympic Movement explicitly sets out to create a more equitable society and promote participation for all and fairness in disability sport. This is primarily achieved through the use of a range of interventions with less attention given to how economic factors may hinder access and achievement in Paralympic sport. We investigated how country-level economic variables influence the level of participation and achievement in the 2015 International Paralympic Committee (IPC) Athletics Championships held in Doha. We used multiple regression analysis to show how levels of participation and achievement in the Championships were significantly determined by economic factors independent of population size.

Our data show that in spite of the ideals of inclusion and fairness within the Paralympic Movement and the considerable effort expended on the use of technologies to achieve this, economic factors continue to exert a statistically significant influence on both the level of participation and achievement of Paralympic athletes. LMICs participate at lower levels and achieve fewer medals when compared to HICs. These differences are particularly marked in events that have a high cost of participation. Our findings raise questions regarding the use of current technologies and the level to which they are able to truly disrupt the politics of global inequality in sport.

## Introduction

In aiming for global fairness and inclusivity for all people of the world, and a “better world” for all people with disabilities, the Paralympic movement aligns itself with global international initiatives regarding fairness and inclusivity, such as the UN Convention on the Rights of Persons with Disabilities [1] and the World Health Organization/World Bank World Disability Report [2]. The International Paralympic Committee (IPC) explicitly states that its aim is to “make for a more inclusive society” (para. 5) [3] and “to increase inclusion by breaking down social barriers and discrimination towards people with an impairment” (para. 9) [3]. This is a very ambitious goal for any sporting body, and sets the bar high for what competitive sport can and cannot achieve in the broader society. It begs the question: how does the IPC, as a leader in the world of para-sport, enact and contribute to fairness and inclusion globally?

This question has been considered from a disability activism perspective in the UK [4], and the answers are clearly complex. It cannot be addressed in a single small study, but as part of a larger project exploring issues associated with sports opportunities for people with disabilities. In this paper we use data from a recent major Paralympic event–the 2015 IPC Athletics World Championships held in Doha, Qatar from 21 to 31 October 2015 –to explore questions regarding disability inclusion in sport. Specifically, we explore factors which determine both participation and success in the Paralympic sport of athletics (track and field). If Paralympic sport is centrally about fairness and inclusion, to what extent do the data on participation and achievement in Doha demonstrate a disruption of the pervasive global power and resource inequalities that exclude people with disabilities from opportunities to participate fully in society?

## Fairness and the Paralympics

The values of fair competition and intolerance of the use of technologies to obtain an advantage over other athletes are central the principles that govern international sport. Central to these values are concerns such as doping in sport and other means by which athletes are considered to achieve an unfair advantage. For example, there was an outcry when the South African runner, Caster Semenya, was subjected to gender testing following her win at the 2009 IAAF World Championships [5]. Similarly, there was considerable controversy on whether to allow Paralympian Oscar Pistorius to run alongside able-bodied competitors, with some questioning whether his sports prostheses gave him an unfair advantage [6].

In general, concepts of fairness in sport are linked to ideas about the training and development of the body for competition without the use of technologies which have the potential to transform an athlete into something other than the best version of his or herself in a “natural” state. Sporting prowess is commonly linked to ideas about moral worth, with good sportsmanship being viewed as the enactment of fairness and the eschewing of unfair advantage. This value of fairness is central to the Paralympic movement. That said, the Paralympics, in its own pursuit of fairness, readily utilizes extraneous technologies in order to promote fairness. There are essentially four kinds of interventions (or what in science and technology studies are known as “technologies”) essential to Paralympism’s pursuit of fairness for athletes with disabilities. First, there is a complex and developing technology of categorisation, whereby an attempt is made to order and categorise different types of bodies into classes in which people with atypical bodies can compete fairly against those with similar, or functionally equivalent, atypicalities. Second, there are technical adaptations of the ways in which sports are performed to ensure that athletes with disabilities can execute them–for example, the “two bounce” rule in wheelchair tennis whereby in competitions between able-bodied and wheelchair-using players, the wheelchair users may have the ball bounce twice as opposed to once for able-bodied players Third, there are technologies of assistance, whereby athletes with impairments may make use of human assistants in order to compete, as in the case of blind athletes who are accompanied by sighted assistants as they race. Fourth, there are material technologies, the use of which enable athletes to compete. These technologies include a range of assistive devices such as sports prostheses, wheelchairs and throwing frames in field events.

Through the application of these technologies, the Paralympic movement has created many and varied opportunities for people with disabilities to participate in and excel at sport. For example, on the question of classification, the IPC “Layman’s Guide to Paralympic Classification” states:

Classification provides a structure for competition. Athletes competing in Paralympic sports have an impairment that leads to a competitive disadvantage in sport. Consequently, a system has to be put in place to minimize the impact of impairments on sport performance and to ensure the success of an athlete is determined by skill, fitness, power, endurance, tactical ability and mental focus. This system is called classification. (p. 1) [7]

The unique systems of Classification used in Para-sport perform two critical functions to support the realisation of this vision. They: (1) define who is eligible to compete in para-sport and consequently has the opportunity to reach the goal of becoming a Paralympic athlete; and (2) group athletes into sport classes which aim to ensure that the impact of impairment is minimised and sporting excellence determines which athlete or team is ultimately victorious. It is important to note that the competitive structure provided by classification systems is not only important for elite sport but also is essential for promoting grassroots participation in para-sport by people with an impairment [8].

Here, it can be seen that the Paralympic system shares with other sporting codes the goal of ensuring that “skill, fitness, power, endurance, tactical ability and mental focus” (p. 1) [7] are what determine sporting excellence. However, the Paralympic system goes further than this. Para-athletes are called upon to “inspire and excite the world” (p. 1) [7]. Paralympism, furthermore, strives “to make for a more inclusive society for people with an impairment through para-sport” (para. 5) [3], and it is also claimed that “through sport para-athletes challenge stereotypes and transform attitudes, helping to increase inclusion by breaking down social barriers and discrimination towards people with an impairment” (para. 9) [3].

Para-athletes are also called upon to “inspire” (para. 2) [3] others primarily through their use of their “skill, fitness, power, endurance, tactical ability and mental focus” (p. 1) [7] to overcome physical barriers which may appear all but insurmountable to others. The use of disabled people as players in what some have termed “inspiration porn” is highly criticised in some contemporary disability studies circles [5, 9, 10]. On the other hand, the recognition by the IPC that attitudinal barriers are central to the exclusion of many disabled people from the social mainstream has much in common with contemporary views of disability, which place environmental and attitudinal barriers at the centre of theorising about social exclusion and denial of access to full participation in society [11].

There is common cause between Paralympism on the one hand and emancipatory disability activism on the other; both movements agree that attitudes towards disabled bodies must change. However, Paralympism and emancipatory disability activism are partially at odds with each other in terms of the means that should be used to effect these attitudinal changes. Disability activists argue that society in general must be more accepting of a wide range of bodies and abilities. It is not acceptable for disabled people to be “dustbins for disavowal,” as stated by disability scholar Tom Shakespeare [12]. Proponents of the social value of Paralympism would concur with this view, but would argue that a key way to achieve attitudinal change is by demonstrating that bodies which may be generally thought to be deficient can, commonly through the judicious application of appropriate technologies, be shown to be surprisingly (and inspiringly), more able than what might have been thought. Through applying appropriate technologies to the body, in other words, the Paralympic ideals can be seen to enable a more equal, fair, and inclusive world.

How are fairness, equity and inclusivity understood in this context of the Paralympic Movement? First, as we have suggested, there is an emphasis on applying technologies to the body in order to optimize the function of individual athletes who are appropriately assisted and classified. Second, however, there is also a much broader global claim. Arguably, in this regard, the 2012 London Paralympic Games marked a watershed moment in the realization of the Paralympic Movement’s ideals [13]. It was the biggest, most accessible and best-attended competition in the 64-year history of the event [13]. The London Games had as an explicit aim to contribute to “a better world for all people with a disability” [13, 14]. The vision of Paralympic sport, therefore, extends far beyond the bodies of athletes from around the world, into the realm of global fairness for all people with disabilities. This is an especially important aim to explore, as it is well established that both the number and the proportion of people with disabilities in the general population are greater in lower income countries than in more wealthy countries (the only exception being the fact of there being proportionally more people who survive into advanced age in wealthy countries, and ageing-associated disabilities are therefore higher in countries with higher life expectancies) [2].

The IPC is clear about its contribution to global equality. For example, in its online training module, “Introduction to the Paralympic Movement”, the following statement is made:

The Paralympic Movement builds a bridge which links sport with social awareness thus contributing to the development of a more equitable society with respect and equal opportunities for all individuals. (p. 6) [15]

Clearly, the notion of a “more equitable society” has an economic component as well as a participatory component–the latter addressing the question of the extent to which all people have maximal opportunities to participate in all aspects of society, including participation in sport. In this article we review data from the 2015 Doha IPC Athletics World Championships to explore how equality and inequality are enacted in this context, and we shall show that the data from the Doha event raise questions at the nexus between economic status and equity in participation. The purpose of this study therefore was to determine the current context of the Paralympic Movement and how it does or does not contribute to a more equitable society.

## Methods

We investigated how country-level economic variables influence the level of participation and achievement in the 2015 IPC Athletics World Championships held in Doha. We were interested in determining the extent to which a country’s level of participation and achievement at the championships were a function of population size, level of economic development and Gross Domestic Product. Furthermore, we wanted to establish whether the economic determinants of participation and achievement were more marked for female athletes than males, because of the history of research showing gender biases in relation to disability [12].

### Data collection

We extracted the following data from the 2015 IPC Athletics World Championship programme:

1. List of participating countries;
2. Level of participation for each country by gender, as measured by the total number of participants from each country in each event (i.e., the number of person-participation events); and
3. Level of achievement for each country by gender, as measured by the total number of medals earned by each participating country.

We also compiled a list of non-participating countries. In addition, we extracted the following data for all participating and non-participating countries from the World Bank website:

1. Economic classification (i.e., High Income Country, Middle Income Country and Low Income Country). For the purposes of analysis High Income Countries (HICs) were compared to Low and Middle Income Countries (LMICs);
2. Per capita Gross Domestic Product (per capita GDP); and
3. Population size.

### Data analysis

Data were entered into SPSS and multiple regression analysis was performed in order to establish if: (1) there was a statistically significant difference between the level of economic development and whether or not a country participates in the championships; (2) independent of population size, level of participation (measured by number of person-participation events) is dependent on a country’s level of economic development (measured by the World Bank system of economic classification and per capita GDP); (3) if the associations between level of participation and level of economic development are stronger for females than for males; (4) independent of population size, level of achievement (measured by number of medals earned) is dependent on a country’s level of economic development (measured by the World Bank system of economic classification and per capita GDP)

All the relevant variables were extremely positively skewed. After deleting countries like China, India, Russia and USA, which were outliers in terms of population size, the variables were still very positively skewed. Logarithmic data transformation was then performed on the skewed data. This improved the situation to such an extent that, although the variables were still positively skewed, the assumptions for doing regression analyses (the appropriate method to answer the research questions and control for population size), were met.

## Findings

A total of 96 countries participated in the championships of which 52% were High Income Countries (HIC), 43.75% Middle Income Countries (Upper Middle Income and Lower Middle Income, according to World Bank Classification) (MIC) and 4.1% were Low Income Countries (LIC). A total of 116 countries did not participate, 25% of which were HIC compared to 51.72% MICs and 23.29% LICs. As shown in Table 1, 52.8% (n = 50) of all HIC countries who could potentially have participated took part in the championships. HICs were 3.4 times more likely than Low or Middle Income Countries to participate in the Championships (OR 3.26, 95% CI 1.82–5.83, p < .0001).

**Table 1:** Proportion of Countries by World Bank classification that participated in the Championships.

	Participated		Did not participate		Total
	N	%	N	%	
High Income	50	52.08%	29	25.00%	79
Upper Middle Income	26	27.08%	27	23.28%	53
Lower Middle Income	16	16.67%	33	28.45%	49
Low Income	4	4.17%	27	23.28%	31

### Levels of participation as a function of economic variables

There was a significant positive correlation between GDP and the total level of athlete participation per country calculated as number of person-participation events (r (190) = .445, p < .001, r^2^ = .198). A series of multiple regression analyses were performed. Population size was entered into each regression model as the first step, with economic classification (HIC versus LMICs) and GDP entered into the model as the second step to determine: (1) if a significant amount of additional variance in the level of participation can be explained by economic classification or GDP over and above the variance explained by population size; and (2) if economic classification or GDP significantly predicts the level of participation when population size is held constant. The same analyses were performed independently for both males and females.

From the results in Table 2, it is evident that a significant amount of additional variance in the total level of participation is explained by economic classification (8.0%), as well as by GDP (18.9%), over and above the variance explained by population size. Both economic classification (β = .297) and GDP (β = .462) are significant predictors of the total level of participation when population size is held constant. Higher economic classification and higher GDP are associated with higher total levels of participation.

**Table 2:** Results of multiple regression analyses predicting total level of participation (total number of participants) by economic classification (EC) and GDP (95% confidence intervals for *b* in parentheses).

EC	*b*	*SE b*	*β*	*P*
Step 1				
Constant	1.262			
Population size	0.011	0.141		
	(0.007–0.015)	0.002	.482	.001
Step 2				
Constant	0.978	0.161		
Population size	0.013	0.002	.572	.001
	(0.009–0.017)			
EC^a^	0.314	0.099	.297	.002
	(0.117–0.510)			
GDP				
Step 1				
Constant	1.262	0.141		
Population size	0.011	0.002	.482	.001
	(0.007–0.015)			
Step 2				
Constant	0.273	0.224		
Population size	0.014	0.002	.641	.001
	(0.010–0.018)			
GDP^b^	0.070	0.013	.462	.001
	(0.044–0.097)			

a. ΔR^2^ = .080, ΔF (1, 86) = 10.060, p = .002

b. ΔR^2^ = .189, ΔF (1, 86) = 28.010, p = .001

From the results in Table 3, it is evident that for males, a significant amount of additional variance in the level of participation is explained by economic classification (3.9%), as well as by GDP (10.5%), over and above the variance explained by population size. Both economic classification (β = .207) and GDP (β = .345) are significant predictors of level of participation when population size is held constant. Higher economic classification and higher GDP are associated with higher levels of male participation.

**Table 3:** Results of multiple regression analyses predicting level of participation for males (number of male participants) by economic classification (EC) and GDP (95% confidence intervals for b in parentheses).

EC	*b*	*SE b*	*β*	*p*
Step 1				
Constant	1.125	0.145		
Population size	0.009	0.002		
	(0.005–0.014)		.428	.001
Step 2				
Constant	0.927	0.172		
Population size	0.011	0.002	.490	.001
	(0.006, 0.015)			
EC^a^	0.219	0.105	.207	.041
	(0.009–0.428)			
GDP				
Step 1				
Constant	1.125	0.145		
Population size	0.009	0.002	428	.001
	(0.005, 0.014)			
Step 2				
Constant	0.387	0.248		
Population size	0.012	0.002	.546	.001
	(0.008–0.016)			
GDP^b^	0.052	0.015	.345	.001
	(0.023–0.082)			

a. ΔR^2^ = .039, ΔF (1, 86) = 4.315, p = .041

b. ΔR^2^ = .105, ΔF (1, 86) = 12.684, p = .001

From the results in Table 4, it is evident that, for females, a significant amount of additional variance in the level of participation is explained by economic classification (10.0%), as well as by GDP (16.9%), over and above the variance explained by population size. Both economic classification (β = .332) and GDP (β = .437) are significant predictors of level of participation when population size is held constant. Higher economic classification and higher GDP are associated with higher levels of female participation. The influence of economic classification and higher GDP was higher for females than for males (by comparison of the R^2^ in Tables 3 and 4).

**Table 4:** Results of multiple regression analyses predicting level of participation for females (number of female participants) by economic classification (EC) and GDP (95% confidence intervals for *b* in parentheses).

EC	*b*	*SE b*	*β*	*p*
Step 1				
Constant	0.623	0.226		
Population size	0.009	0.003	.289	.006
	(0.003, 0.016)			
Step 2				
Constant	0.160	0.257		
Population size	0.013	0.003	.389	.001
	(0.006–0.019)			
EC^a^	0.513	0.158	.332	.002
	(0.199–0.827)			
GDP				
Step 1				
Constant	0.623	0.226		
Population size	0.009	0.003	.289	.006
	(0.003–0.016)			
Step 2				
Constant	0.746	0.372		
Population size	0.014	0.003	.439	.001
	(0.008, 0.021)			
GDP^b^	0.097	0.022	.437	.001
	(0.053–0.141)			

a. ΔR^2^ = .100, ΔF (1, 86) = 10.544, p = .002

b. ΔR^2^ = .169, ΔF (1, 86) = 19.411, p = .001

### Levels of achievement as a function of economic variables

A series of multiple regression analyses were again performed. Population size was entered into each regression model as the first step, with economic classification (HIC versus LMIC) and GDP entered into the model as the second step to determine: (1) if a significant amount of additional variance in the level of achievement can be explained by economic classification or GDP over and above the variance explained by population size; and (2) if economic classification or GDP significantly predicts the level of achievement when population size is held constant. The same analyses were performed independently for both males and females.

From the results in Table 5, it is evident that a significant amount of additional variance in the total level of achievement is explained by economic classification (4.8%), as well as by GDP (12.1%), over and above the variance explained by population size. Both economic classification (β = .230) and GDP (β = .370) are significant predictors of the total level of achievement when population size is held constant. Higher economic classification and higher GDP are associated with higher total level of achievement.

**Table 5:** Results of multiple regression analyses predicting total level of achievement (total number of medals won) by economic classification (EC) and GDP (95% confidence intervals for *b* in parentheses).

EC	*b*	*SE b*	*β*	*p*
Step 1				
Constant	0.303	0.220		
Population size	0.011	0.003	.353	.001
	(0.005–0.018)			
Step 2				
Constant	0.019	0.259		
Population size	0.014	0.003	.423	.001
	(0.007–0.020)			
EC^a^	0.355	0.159	.230	.028
	(0.040–0.671)			
GDP				
Step 1				
Constant	0.303	0.220		
Population size	0.011	0.003	.353	.001
	(0.005–0.018)			
Step 2				
Constant	0.854	0.373		
Population size	0.016	0.003	.480	.001
	(0.009–0.022)			
GDP^b^	0.082	0.022	.370	.001
	(0.038–0.126)			

a. ΔR^2^ = .048, ΔF (1, 86) = 5.015, p = .028

b. ΔR^2^ = .121, ΔF (1, 86) = 13.784, p = .001

From the results in Table 6, it is evident that for males, a significant amount of additional variance in the level of achievement is explained by GDP (10.1%) over and above the variance explained by population size. Economic classification, however, does not explain a significant amount of additional variance in the level of achievement. Only GDP (β = .338) is a significant predictor of level of achievement when population size is held constant. Higher GDP is associated with higher levels of male achievement.

**Table 6:** Results of multiple regression analyses predicting level of achievement for males (number of medals won by male participants) by economic classification (EC) and GDP (95% confidence intervals for *b* in parentheses).

EC	*b*	*SE b*	*β*	*p*
Step 1				
Constant	0.282	0.205		
Population size	0.009	0.003	0.310	.003
	(0.003–0.015)			
Step 2				
Constant	0.033	0.244		
Population size	0.011	0.003	.368	.001
	(0.005–0.017)			
EC^a^	0.275	0.149	.194	.069
	(-0.022–0.572)			
GDP				
Step 1				
Constant	0.282	0.205		
Population size	0.009	0.003	.310	.003
	(0.003–0.015)			
Step 2				
Constant	0.689	0.354		
Population Size	0.013	0.003	.426	.001
	(0.007–0.019)			
GDP^b^	0.069	0.021	.338	.001
	(0.027–0.111)			

a. ΔR^2^ = .034, ΔF (1, 86) = 3.392, p = .069

b. ΔR^2^ = .101, ΔF (1, 86) = 10.810, p = .001

From the results in Table 7, it is evident that, for females, a significant amount of additional variance in the level of achievement is explained by economic classification (7.1%), as well as by GDP (13.5%), over and above the variance explained by population size. Both economic classification (β = .278) and GDP (β = .392) are significant predictors of level of achievement when population size is held constant. Higher economic classification and higher GDP are associated with higher levels of female achievement, and this is stronger for females than for males.

**Table 7:** Results of multiple regression analyses predicting level of participation for females (number of medals won by female participants) by economic classification (EC) and GDP (95% confidence intervals for *b* in parentheses).

EC	*b*	*SE b*	*β*	*p*
Step 1				
Constant	0.051	0.206		
Population size	0.009	0.003	.304	.004
	(0.003–0.015)			
Step 2				
Constant	0.306	0.239		
Population size	0.012	0.003	.387	.001
	(0.005–0.018)			
EC^a^	0.394	0.146	.278	.009
	(0.103–0.685)			
GDP				
Step 1				
Constant	0.051	0.206		
Population size	0.009	0.003	.304	
	(0.003–0.015)			
Step 2				
Constant	1.073	0.347		
Population size	0.013	0.003	.438	.001
	(0.007–0.019)			
GDP^b^	0.080	0.021	.392	.001
	(0.039, 0.121)			

a. ΔR^2^ = .071, ΔF (1, 86) = 7.245, p = .009

b. ΔR^2^ = .135, ΔF (1, 86) = 15.089, p = .001

### Achievement and participation as a function of cost of participation

We considered the costs of participation for all track and field events based on whether or not the athletes required human assistance (as in the case of visually impaired runners who require guides) or specialist equipment (such as prosthetics or throwing frames). We calculated the costs of participation for each class of event and divided them into three groups; those with a low cost of participation, those with a moderate cost of participation and those with a high cost of participation. We then calculated each country’s level of participation (the total number of athletes competing) in the low, moderate and high cost groups. Similarly, we determined each country’s level of achievement (the total number of medals earned) in the low, moderate and high cost groups. We then used a nonparametric t-test to determine if the level of participation and level of achievement in each of the low, moderate and high cost groups was significantly different for HICs when compared to LMICs. The results of this statistical analysis is shown in Tables 8 and 9 below. Our analysis confirmed the hypothesis that while the level of participation and achievement for HICs and LMICs is not significantly different in events which have a low or moderate cost of participation, there is a significant difference in events with a high cost of participation. HICs are statistically more likely than LMICs to participate in events which have a high cost of participation (p = 0.0231) and are statistically more likely to win medals in these events (p = 0.0197).

**Table 8:** Level of achievement for HICs compared to LMICs for events which have a low cost of participation, moderate cost of participation and high cost of participation.

	T value	df	P value
Events with a low cost of participation	t = 0.9858	df = 93	0.3268
Events with a moderate cost of participation	t = 0.8540	df = 93	0.3953
Events with a high cost of participation	t = 2.3092	df = 93	0.0231*

**Table 9:** Level of achievement for HICs compared to LMICs for events which have a low cost of participation, moderate cost of participation and high cost of participation.

	T value	df	P value
Events with a low cost of participation	t = 0.5554	df = 93	0.5800
Events with a moderate cost of participation	t = 0.3399	df = 93	0.7347
Events with a high cost of participation	t = 2.3727	df = 93	0.0197*

## Discussion

Our study has important limitations that need to be considered before we discuss our data. Firstly these data are generated from only one Paralympic sport, namely athletics (track and field) which may have a different profile to other Paralympic sports—for example—goalball or sitting volleyball, where technologies may play a less prominent role. Furthermore the Doha athletics World Championships were held in a country and venue which is expensive; there may therefore be some bias of the sample and indeed the results of this study might have been different if it the Championships were held in a low or middle income country. In this regard, it is noteworthy that organisers of the competition chose to have the event at a venue which incurs high costs to the athletes and countries participating. The potential impact of this decision on global access and equality may likely have been anticipated.

Our data reveal that levels of participation and achievement in the 2015 IPC Athletics World Championships are significantly determined by economic factors, independent of population size. For both males and females, a significant amount of additional variance in the level of participation is explained by economic classification as well as by GDP, over and above the variance explained by population size. Also, for both males and females, economic classification and GDP are significant predictors of level of participation when population size is held constant. Looking at the percentages of additional variance explained and the standardised beta coefficients, the values for females are higher than for males, indicating that economic factors are more significant predictors of participation rates for females when compared to males.

Furthermore, a significant amount of additional variance in the level of achievement for both males and females is explained by GDP over and above the variance explained by population size. For both males and females, GDP is a significant predictor of level of achievement when population size is held constant, while economic classification is only a significant predictor for females. Looking at the percentages of additional variance explained and the standardised beta coefficients, values are higher for females than for males. These data indicate that the level of achievement in the championships was significantly determined by economic factors, with the association being strongest for female athletes.

Our data are in accordance with other studies which have shown that success (medals) in Olympic sport is also closely related to GDP and team size (which is indirectly related to country wealth [16, 17]. However, most of these studies have noted that Olympic success is also related to population size and influenced significantly by public investment. [18]. To the best of our knowledge, the current study is the first to describe that success in Paralympic sport is related to GDP independent of population size.

Our data reveal that in spite of the ideals of inclusion and fairness within the Paralympic Movement and the considerable effort expended on the use of technologies to achieve fairness and inclusion, economic factors continue to exert a statistically significant influence on both level of participation and achievement of Paralympic athletes. LMICs participate at lower levels and achieve fewer medals when compared to HICs. These differences are particularly marked in events which have a high cost of participation.

What do these data say about the extent to which the Paralympic Movement is achieving its goals of global fairness and inclusivity for all people of the world, and a “better world” for all people with disabilities? These data suggest that levels of participation and achievement for athletes with disabilities from LMICs are lower than those of their counterparts living in the developed world. As noted, there is considerable focus on the use of technologies to promote inclusion and fairness. Some of these technologies cost money, and it would seem that economic factors continue to exert a significant influence not only on who gets to take part in Paralympic competitions but also on who wins medals at these events. To an extent, participation rates at major Paralympic events do demonstrate an improvement on expected rates of participation. As we noted, HICs were 3.4 times more likely to participate in the Doha World Championships, however World Bank Data suggest that the median GDP per capita in HICs is approximately 11 times higher than in LMICs [19].

In spite of the significant influence of economic factors on both participation and achievement at the Doha World Championships, insufficient attention has been given to what should be done to promote inclusion and level the playing fields for athletes with disabilities in LMICs. If the Paralympic movement is serious about its goal of achieving fairness and inclusion for all persons with disabilities, it will need to give attention to the structural and economic factors within LMICs that hinder participation and achievement. The application of technologies to the bodies of athletes in conditions where there is relative economic prosperity may well serve to equalise opportunities within higher income contexts. These technologies cannot, however, solve the global problem of access to sporting opportunities in LMIC.

While there is an explicit acknowledgment within the global Paralympic movement of the role of social and attitudinal forces in limiting opportunities for persons with disabilities, there is currently insufficient acknowledgment of the role played by economic factors in limiting opportunities for participation in disability sport and in promoting achievement. The high ideals and inspirational tone of the ideologies of Paralympism have merit, but have been at times criticised for the ways in which they obscure the realities of the lives of many disabled people who are not elite athletes. They also may obscure the difficult global reality of unequal resources and the impact that global inequality has on disability and participation.

These findings raise questions regarding what may be done to achieve global fairness and minimise the impact of economic factors on levels of participation and achievement in Paralympic sport. One possibility is to limit the use of expensive technologies. This has, for example, been done with throwing frames in some athletic events. Furthermore, the recent introduction of lower cost wheelchairs and racing wheelchairs as an initiative catalysed by the IPC Agitos Foundation is an important step in the right direction. [19] Another possibility is to consider critically how the rules, adaptations and classification system might inadvertently raise the costs of participation and thus exacerbate economic inequalities in levels of achievement. Clearly, this is not an easy problem to solve in a way that is sustainable. It is important to acknowledge efforts already made and being made, notably through the proactive work of the Agitos Foundation and other targeted development initiatives currently operated by the IPC [20]. It is the authors’ hope is that the analysis herein will assist with efforts of the Paralympic movement to focus more fully on global equity issues.

## Conclusion

It is clearly not the role, nor should it be, of the IPC to tackle inequality on a global scale–no sporting body can do this, and it is important to recognise the work the IPC has already done in this regard. However, the inspirational and aspirational tone of language used throughout the Paralympic movement may lead to an expectation that fairness and equality are enacted through Paralympic sport to an extent which is not the case. The data we have used in this article represent a small and limited slice of possible data which could be analysed in this regard. We have not, for example, studied the extent to which Paralympic sport is professionalised in wealthier countries, offering greater access to opportunities and skills development for talented Paralympians than is available to athletes in less wealthy countries. More research is needed in areas such as this.

We do not believe that it is wrong for any sporting body to have aspirations of contributing to a better and more equitable world. However, for equality to be achieved it is important that the scope be broadened away from technologies of the body to the more complex and difficult task of contributing to global social justice. This requires more than rhetoric, more than inspiration, and a concrete investment of resources in low resource contexts. We make these comments as strong supporters of the Paralympic movement, noting that Paralympic sport has done a great deal to improve perceptions of people with disabilities and what they can achieve. There is, however, an important challenge herein, and certainly not just for the IPC, but also for athletes, coaches, and administrators throughout the movement. It is a human rights issue that all people with disabilities, not just elite athletes, and not just those in wealthier countries, are given the opportunity to participate fully in physical activity and sport [21]. In this regard, the Paralympic movement does play an important role in potentially addressing much broader concerns regarding the problem of global inequality.

## Supporting Information

S1 Table Gender and participation. Spreadsheet showing male and female participants in track and field events + countries represented + medals obtained by person and country
(XLSX)

S2 Table Participation and socioeconomic status. **S**preadsheet showing participating countries' socio-economic statistics as made available by the World Bank
(XLSX)

S3 Table Medals by country: track events. **S**preadsheet showing number of participants by each country + gold, silver, and bronze medals obtained by each country—for track events only
(XLSX)

S4 Table Medals by country: field events. **S**preadsheet showing number of participants by each country + gold, silver, and bronze medals obtained by each country—for field events only
(XLSX)

S5 Table Non-participation and socioeconomic status. **S**preadsheet showing non-participating countries' socio-economic statistics as made available by the World Bank
(XLSX)

S6 Table Data for analysis: medals. **S**preadsheet showing data for analysis–medals
(XLSX)

S7 Table Data for analysis: participation. **S**preadsheet showing data analysis for participation
(XLSX)
